# O-50 AN OUTBREAK OF PANTON-VALENTINE LEUCOCIDIN-PRODUCING METHICILLIN-RESISTANT STAPHYLOCOCCUS AUREUS SKIN AND SOFT TISSUE INFECTION IN CHILDREN ASSOCIATED WITH BOUNCY CASTLE USE IN EASTERN IRELAND IN 2025

**DOI:** 10.1093/pubmed/fdag046.044

**Published:** 2026-07-09

**Authors:** Dr Domhnall McGlacken-Byrne, Dr Gráinne Brennan, Dr Eimear Kitt, Dr Keith Ian Quintyne, Dr Eimear Brannigan, Dr Máirín Boland, Ms Andrea King, Dr Michael Barrett, Dr Noelle O’Loughlin, Dr Desmond Hickey

**Affiliations:** Health Service Executive (HSE) Department of Public Health, Dublin and Midlands; National MRSA Reference Laboratory; Children's Health Ireland at Crumlin; National Health Protection Office, Health Service Executive (HSE); Antimicrobial Resistance and Infection Control Team, Health Service Executive (HSE); Health Service Executive (HSE) Department of Public Health, Dublin and Midlands; Health Service Executive (HSE) Department of Public Health, Dublin and Midlands; Children's Health Ireland at Crumlin; Health Service Executive (HSE) Department of Public Health, Dublin and Midlands; Health Service Executive (HSE) Department of Public Health, Dublin and Midlands

## Abstract

**Background:**

We describe an outbreak of Panton-Valentine leucocidin (PVL) producing community-acquired methicillin-resistant Staphylococcus aureus (CA-MRSA) in children in eastern Ireland, associated with bouncy castle use.

**Objectives:**

To describe the findings, management strategies and insights arising from a large and complex CA-MRSA outbreak among children.

**Methods:**

A regional department of public health was notified in autumn 2025 of several hospital presentations by children with skin and soft tissue infection (SSTI), associated with attendance at a recent community social gathering. An outbreak was declared. Phone interviews were conducted with a parent or guardian of each case, obtaining demographic, clinical and exposure information. Case definitions were drafted and iteratively refined.

**Results:**

A total of 48 children and no adults were identified as cases; of these, 47 attended the same outdoor social gathering in August 2025 which featured bouncy castle play. Most cases developed skin symptoms within two or three days of the gathering. The commonest affected body site was the legs, particularly the thighs. Thirty-three children were treated by their general practitioner (GP). A further 15 attended a hospital emergency department or day ward, of whom 4 were hospitalised, with no deaths. All confirmed cases exhibited an identical strain type, t016-ST22-MRSA-IV, which was PVL-producing. This strain has been described as hypervirulent but is rare in Ireland. The causal isolate exhibited a distinctive antibiogram. An epidemiological curve was created, which was suggestive of a point source outbreak. Infection control measures were enacted, including treatment and decolonisation of cases; decolonisation of asymptomatic household contacts; and communication with affected families and stakeholders such as the bouncy castle providers.

**Conclusions:**

This outbreak highlights the risk of CA-MRSA outbreaks in contexts involving prolonged, close physical contact, such as play involving bouncy castles. Infection control measures to mitigate transmission in the community are critically important to control such outbreaks.

